# *Pichia anomala* Induced With Chitosan Triggers Defense Response of Table Grapes Against Post-harvest Blue Mold Disease

**DOI:** 10.3389/fmicb.2021.704519

**Published:** 2021-07-22

**Authors:** Esa Abiso Godana, Qiya Yang, Lina Zhao, Xiaoyun Zhang, Jizhan Liu, Hongyin Zhang

**Affiliations:** ^1^School of Food and Biological Engineering, Jiangsu University, Zhenjiang, China; ^2^School of Agricultural Engineering, Jiangsu University, Zhenjiang, China

**Keywords:** grape, post-harvest disease, *Pichia anomala*, *Penicillium expansum*, enzyme activity, total phenol and flavonoid

## Abstract

To study the mechanism by which *Pichia anomala* induced with chitosan (1% w/v) controls blue mold disease in table grapes caused by *Penicillium expansum*, this study evaluated alterations in three yeast enzymatic activities. The changes in the five primary disease defense-related enzymes and two non-enzyme activities of table grapes were assayed. The results of the study showed that chitosan (1% w/v) significantly increased the yeast β-1,3-glucanase, catalase (CAT), and malondialdehyde (MDA) activities. Furthermore, *P. anomala* alone or induced with chitosan (1% w/v) significantly increased the table grapes enzymatic activities of Polyphenol oxidase (PPO), phenylalanine (PAL), peroxidase (POD), and catalase (CAT) compared to the control. The RT-qPCR results also confirmed that the genes of these major disease defense enzymes were up-regulated when the table grapes were treated with *P. anomala*. The highest results were recorded when the fruit was treated by yeast induced with chitosan (1% w/v). The phenolic compounds, in addition to their nutritional value, can also increase the antimicrobial properties of table grapes. The current experiment determined that the total phenol and flavonoid contents of table grapes showed the highest results for fruits treated by *P. anomala* induced with chitosan compared with the control. Generally, the increment of these fruit enzymatic and non-enzymatic activities shows improved table grape defense against the pathogenic fungus. The induction of the yeast with chitosan also increases its bio-control efficacy against the pathogen. This study will enable future detailed investigation in the yeast pathogen control mechanisms and the use of yeasts as bio-pesticides.

## Introduction

The United Nations has indicated that for the first time in recent history world hunger is on the rise. It is clear that we are not going to be able to meet the global food shortage crisis by simply producing more food as we did during the “Green Revolution.” Even with our most advanced food production technologies, we are going to fall far short of producing enough food to feed the world’s increasing population, which is expected to reach 9.6 billion by 2050. To feed this growing population, we must save substantially more food than we currently produce. Until recent years, a disproportionate amount of our resources have been invested in the production of food (95%) while only (5%) has been dedicated to the post-harvest preservation of food. This has left us with tremendous post-harvest “Skill Gaps” and “Technology Gaps” in developing countries.

Of the total food produced for human consumption in the world, one-third is lost or wasted every year. This amounts to an approximate cost of $680 billion in developed countries and around $310 billion in developing countries (Sawicka, 2019). When we compare industrialized and developing countries, a similar amount of food is wasted (670 and 630 million tons, respectively). Of this massive world food loss, fruits and vegetables take the largest share. Generally, post-harvest loss of these perishables is estimated from 40 to 50%. Although there are different causes for post-harvest losses for these perishables, post-harvest diseases are major contributing factors. Therefore, the control of post-harvest diseases could significantly help to reduce this massive food loss after production, providing a sustainable solution to increase food availability, reduce pressure on natural resources, eliminate hunger, and improve farmers’ living conditions (Sawicka, 2019).

Due to their physiological nature, table grapes are highly susceptible to post-harvest diseases. *Penicillium expansum* is the primary causal agent of blue mold disease in table grapes (Zoffoli and Latorre, 2011; Sanzani et al., 2013). This destructive pathogen colonizes the fruit surface and causes massive economic loss during storage and shipment (Spadaro and Droby, 2015; Wisniewski et al., 2016). The disease causes light brown skin discoloration initially and ends with soft and wet rot, which quickly colonizes the entire berry (Franck et al., 2005). Blue-green colonies may appear on the fruit’s surface, and the decayed fruit has a musty odor (Zoffoli and Latorre, 2011).

Sulfur dioxide (SO_2_) is an effective treatment that is used to control the table grapes post-harvest decay during cold storage (Sanzani et al., 2012). The fungicide can control the germination and spread of *P. expansum* on the surface of the grapes (Sanzani et al., 2012). However, the use of SO_2_ has some limitations (1) it causes surface bleaching for fresh grapes (de Oliveira et al., 2014), (2) it affects people who have sulfite allergies, and (3) restriction of chemical use in some countries affects grape producers. Therefore, scientists are currently searching for alternative control methods that are safe, effective, and environmentally friendly. Microbial biological control agents are among those getting more attention from researchers in recent years.

Several microorganisms (yeasts, bacteria, and fungi) have been reported for their potential to control post-harvest diseases in fruit and vegetables (Madbouly et al., 2020; Abo-Elyousr et al., 2021). Microorganisms isolated from the surface of fruits have applications due to the high survival rate on surfaces of fruits during treatment. Yeasts that naturally occur on the surface of fruits are useful antagonists for the control of post-harvest diseases. Yeasts are preferred for their ability to survive harsh environmental conditions and because they do not produce mycotoxins and allergies, and survive on a wide range of nutrients (Elisabeth et al., 2002).

The ascomycetous yeast *Pichia anomala* was first reported as having high anti-fungal activity by Boysen et al. (2000). Since then, numerous research on the use of yeast as a potential post-harvest bio-control have been conducted. Our previous research investigated that *P. anomala* has high anti-fungal activity against *P. expansum* in table grapes (Godana et al., 2020). We also determined that the yeast’s pre-treatment with chitosan (1% w/v) enhances the yeast’s antagonistic efficacy. Zhao et al. (2012) reported that the *P. anomala* (strain FY-102) isolated from the surface of apples has a high antagonistic effect against *Botryitis cinerea*. In addition to the control of post-harvest decay of table grapes, *P. anomala* has an outstanding contribution to the viticulture industry. It contributes positively to the wine aroma by the production of volatile compounds, mainly ethyl acetate. It also helps with early fermentation in the wine industry (Renouf et al., 2007). However, excessive use of *P. anomala* can spoil the wine due to there being too much acetic acid and ethyl acetate production (Plata et al., 2003).

In the last three decades, antagonistic yeast and other biological control agents have gained more attention (Wang et al., 2018). Different biological control agents (BCAs), bioactive compounds, food additives, and the mechanisms involved in biological control have been explored to explicate and develop bio-control products. Therefore, understanding the mechanisms of action of BCAs is imperative to improving viability and increasing their efficacy in disease control (Di Francesco et al., 2016).

Numerous reports show that yeasts have the potential to enhance the enzymatic activities and total phenolic compounds during post-harvest treatment in fruits and vegetables. For example, Mahunu et al. (2016) reported that *Pichia caribbica* with phytic acid can induce the PAL, POD, and CAT in apples which help the fruit to combat the blue mold disease. Other researchers also confirmed the use of yeast alone or in combination can significantly induce the major disease defense-related enzymes and phenolic compounds in post-harvest fruits and vegetables (Li and Tian, 2006; Al-Qurashi and Awad, 2015; Qin et al., 2015; Zhao et al., 2018; Abdelhai et al., 2019; Wang et al., 2019).

Our previous study showed a promising result that *P. anomala* could be an alternative post-harvest treatment instead of chemical fungicides to control post-harvest diseases of table grapes. However, the yeast pathogen control mechanism cannot thoroughly be investigated. Studying the yeast’s effect on the fruit physiology, whether it boosts its defense ability is very important for commercializing these antagonistic yeasts in the bio-pesticide market. Therefore, our current study aimed to understand the yeast control mechanisms, specifically (1) to determine changes in the enzymatic activities of *P. anomala* after being incubated in 1% chitosan (w/v), and (2) to ascertain the metabolic response of the enzymatic and non-enzymatic activities of table grapes after being treated by the yeast *P. anomala* induced with chitosan (1% w/v).

## Materials and Methods

### Grapes

Table grapes (*Vitis vinifera* cv. Red Globe) were harvested from a vineyard located in Zhenjiang, Jiangsu, China. Fruits were taken to the research lab and sorted based on their color, size, ripeness, and free from mechanical damage. The sorted fruits were then surface disinfected with 0.2% (v/v) sodium hypochlorite (Tianjin Kaifeng Chemical Co., Ltd.) for 1 min.

### Yeast

*P. anomala* (strain TL0903) was isolated from soil and preserved in the China General Microbiological Culture Collection Center No. 3616 and used for the experiment. The yeast has been preserved in nutrient yeast dextrose agar (NYDA) at 4°C after receiving from the culture center. The yeast was activated in nutrient yeast dextrose broth (NYDB) media before each experiment and was incubated in the rotary shaker (180 rpm) for 24 h at 28°C. The cells were centrifuged at 8000 × *g* for 6 min and washed three times with sterilized distilled water. The cells then adjusted to the required concentration using a hemocytometer (XB-K-250, Jianling Medical Device Co., Danyang, China).

### Chitosan

Chitosan with 90% deacetylation was bought from Sangon Biotech Co., Ltd. (Shanghai, China).

### Pathogen

*Penicillium expansum* was isolated from overripe grapes by our research group according to the method described by Dahiya et al. (2006). After isolation, the fungus was preserved on potato dextrose agar (PDA) media at 4°C. Before each experiment, the fungi were activated, and after 7 days of culture, fresh spore suspension was used. *P. expansum* spores were removed using a bacteriological loop and suspended in sterilized distilled water and thoroughly mixed for 30 min using a vortex. The spore concentration was adjusted to 1 × 10^5^ spores/mL before each experiment.

### Changes in Enzymatic Activities of *P. anomala* Induced With Chitosan

To determine the enzymatic activities of β-1,3-glucanase, catalase (CAT), malondialdehyde (MDA), *P. anomala* was cultured in NYDB media alone or NYDB media supplemented with 1% chitosan. The crude enzyme was extracted according to the methods described by Zhao et al. (2012). Briefly, the culture of yeast alone or incubated with chitosan (1% w/v) was washed three times. Then it was ground in liquid nitrogen and transferred to the 50 mL tubes. The CAT activity was determined according to the method described by Wang et al. (2018). Briefly, 10 mL of phosphate buffer and 10% trichloroacetic acid were added to the ground powder to determine the CAT and MDA activities, respectively. Then the crude extract was used for further analysis. To determine the CAT activity, 10 mL cold sodium phosphate buffer was added to 3 mL crude enzyme extract and centrifuged at 10000 × *g* for 5 min. Then, 0.5 ml of the mixture, 2 mL sodium phosphate buffer, and 0.5 mL hydrogen peroxide were used for the analysis. One unit was defined as the decrease in absorbance 240 nm of 0.01 protein in one minute and was expressed as U/mg protein.

The MDA content was determined according to the method described by Mahunu et al. (2016). Briefly, 2 mL of 0.5 thiobarbituric acid (TBA) in 15% trichloroacetic acid was added to a 1.5 mL crude extract. The mixture was then heated for 20 min at 95°C and then cooled in ice for 5 min. Finally, it was centrifuged at 12000 × *g* for 10 min and the absorbance was measure at 532 and 600 nm. The unit was expressed by μmoL/mg protein. The β-1,3-glucanase activity was analyzed according to the method described by Zhao et al. (2012). Briefly, the culture was collected and centrifuged at 7000 × *g* for 10 min at 4°C. Then, the supernatant was collected and filtered using a 0.45 μL filter membrane. Then 250 μL of crude extract was added to 250 μL of 0.2% laminarin (w/v) in potassium acetate buffer (50 mM, pH 5). The mixture was then incubated at 37°C for 1 h. Finally, 3,5 dinitrosalicylate was added, boiled for 5 min and the absorbance was measured at 500 nm. One unit was defined as the formation of 10 mg glucose by hydrolysis of laminarin in one hour, and the unit was expressed as U/mL supernatant. There were three replications in each treatment and each experiment was repeated two times for confirmation.

### Analysis of Table Grapes Defense-Related Enzyme Activities After Being Treated by *P. anomala* Induced With Chitosan (1% w/v)

#### Crude Extraction

To assess the effect of *P. anomala* alone or induced with chitosan (1% w/v) on major disease defense-related enzymatic activities of table grapes, uniform wounds (3 mm deep × 3 mm diameter) were made at the center of each grape berries. Then, (1) 15 μL sterile distilled water, (2) 15 μL of the *P. anomala* suspension (10^8^ cell/mL), (3) 15 μL of the *P. anomala* suspension (10^8^ cell/mL) pretreated with chitosan (1% w/v) were pipetted into each wound. After 2 h, 15 μL *P. expansum* (1 × 10^5^ spore/mL) was inoculated into each sample. The grapes were then wrapped with plastic film to keep high R.H. (95%) and incubated at 20°C for 3 days. Samples were taken every 24 h for three consecutive days. The wound tissues of five grapes were excised (6 mm deep) using a sterile cork borer. After that, 10 mL of sterile distilled water was added to 3 g of the excised tissue. Then, 50 mM phosphate buffer consisting of polyvinyl pyrrolidone (1%) and 1.33 mM was added to the sample. There were three replications in each treatment and each experiment was repeated two times for confirmation.

#### Polyphenol Oxidase (PPO) Activity

The PPO activity was determined according to Aquino-Bolaños and Mercado-Silva (2004). The crude enzyme extract (0.1 mL) was added to 2.9 mL sodium phosphate buffer (50 mM, pH 6.4, contains 0.1 mM catechol). Then, the samples were heated in a water bath (30°C) for 5 min. Changes in absorbance at 398 nm were recorded every 30 s for 3 min. The enzyme activity was expressed as unit mg/FW.

#### Phenylalanine Ammonia-Lyase (PAL) Activity

The PAL activity was determined according to the method used by Zhang et al. (2013). The crude enzyme (1 mL) was added to a 3 mL borate buffer (50 mM, pH 8.8, containing 10 mM phenylalanine). The solution was thoroughly mixed and heated (37°C for 60 min), and the absorbance was recorded at 290 nm. One PAL unit was defined as the formation of 1 μg of cinnamic acid equivalents per hour, and the specific activity was expressed as unit mg/FW.

#### Peroxidase (POD) Activity

The POD activity was determined according to the method described by Lurie et al. (1997). Crude enzyme extract (0.2 mL) was added to guaiacol 2.2 mL of 0.3% (50 mM sodium phosphate buffer, pH 6.4). The mixture was then incubated at 30°C for 5 min. The POD was measured at 470 nm every 30 s for 3 min. The enzyme activity was expressed as unit mg/FW.

#### Catalase (CAT) Activity

The CAT activity was determined according to the method described by Wang et al. (2004). In detail, 10 mL cold sodium phosphate buffer (100 mM, pH 7.8) was added to 3.0 ml of the crude enzyme extract. The mixture was then centrifuged at 1000 × *g* for 15 min. After that, 2 mL of sodium phosphate buffer (50 mM, pH 7.0), 0.5 mL enzyme extract, and 0.5 mL H_2_O_2_ (40 mM) were used to measure the H_2_O_2_ decomposition at 240 nm absorbance. The CAT enzyme activity was expressed in mg/FW, where one unit of catalase converts one μmol of H_2_O_2_ per min.

### RT-qPCR for Determination of Defense-Related Genes Expression Levels

RNA was extracted from 2 g frozen table grape tissue (stored at −80°C) according to the Spin Column Plant Total RNA Purification Kit (Sangon Biotech, Shanghai, China). Both the purity and quantity were checked using a Spectrophotometer (Thermo Scientific, Fresno, CA, United States) at 260 and 280 nm. The concentration and integrity of RNA were evaluated using the RNA Nano 6000 Assay Kit of the Bioanalyzer 2100 system (Agilent Technologies, Santa Clara, CA, United States). The first-strand cDNA was synthesized from the RNA using the PrimeScript RT reagent kit with gDNA Eraser (Takara-Dalian, China) in PCR System. Specific primers were obtained from Sangon Biotech (Shanghai, China). The RT-qPCR was conducted with Bio-Rad CFX96 Real-Time PCR System (Applied Biosystems, United States) and the computer program set according to Youssef et al. (2020). The RT-qPCR was carried out using T.B. Green^®^ Fast qPCR Mix (TAKARA BIO Inc., Shiga, Japan) and determined in ABI PRISM 7500 Real-Time PCR System (Applied Biosystems, United States) according to the manufacturer’s instructions. The melting cycle was 95°C for 15 s, 60°C for 1 min, 95°C for 15 s, and 62°C for 15 s. Relative gene expression levels were calculated according to the method of 2^–ΔΔCT^ (Livak and Schmittgen, 2001). The experiment was conducted twice, and there were three replications per treatment.

### Non-enzymatic Assays

#### Sample Preparation

The treatments were prepared according to the procedures described above. Samples were then taken at 0, 24, 48, and 72 h for analysis. The wound tissues of table grapes were excised using a sterile cork borer, and 1.0 g of each sample was prepared in 10 mL of 70% ethanol (v/v). After that, the suspension was shaken in a rotary shaker for 2 h (120 rpm at 30°C). Finally, the extract was centrifuged at 1013 × *g* for 5 min, and the supernatant was reserved for further analysis. The suspension was used to determine the total phenolic compound and flavonoid contents (Vázquez et al., 2008).

#### Total Phenolic Content

The total phenolic content of table grapes was measured by spectrophotometry method using the Folin-Ciocalteu reagent (Swain and Hillis, 1959). Specifically, 1 mL extract, 9 mL double-distilled water, and 1 mL Folin-Ciocateau were added to a 25 mL volume flask. The mixture was then shaken for 5 min, and 10 mL Na_2_CO_3_ (7%) were added to the flask. Finally, double distilled water was added to the flask to bring the final volume to 25 mL and incubated for 90 min. Gallic acid at concentrations of 20, 40, 60, 80, and 100 mg/L were used as standard, and the absorption was measured at 750 nm. The results were expressed as mg of gallic acid equivalents (GAE) per 100 g dry weight (DW). The experiment was conducted twice, and there were three replications per treatment.

#### Total Flavonoid Content

The total flavonoid contents of table grapes were determined, according to Gurnani et al. (2016). Briefly, the reaction medium containing 250 μL of the ethanolic extract, NaNO_2_ (5% w/v), AlCl_3_ (10% w/v), and NaOH (4% w/v) was prepared, and absorption was measured at 425 nm against the blank. Quercetin at concentrations of 50, 100, 200, 300, 400, and 500 mg/L were used as standard. The results were expressed as mg quercetin equivalents (QE) per 100 g dry weight (DW). The experiment was conducted twice, and there were three replications per treatment.

### Statistical Analysis

All collected data were from a representative experiment and all experiments were repeated at least twice. The Analysis of Variance (ANOVA) was carried out using Minitab Version 17 (Minitab LLC, State College, PA, United States). Tukey’s test was used for mean comparison, and *P* < 0.05 was considered statistically significant.

## Results

### Changes in Enzymatic Activities of *P. anomala* Induced With Chitosan

**FIGURE 1:**
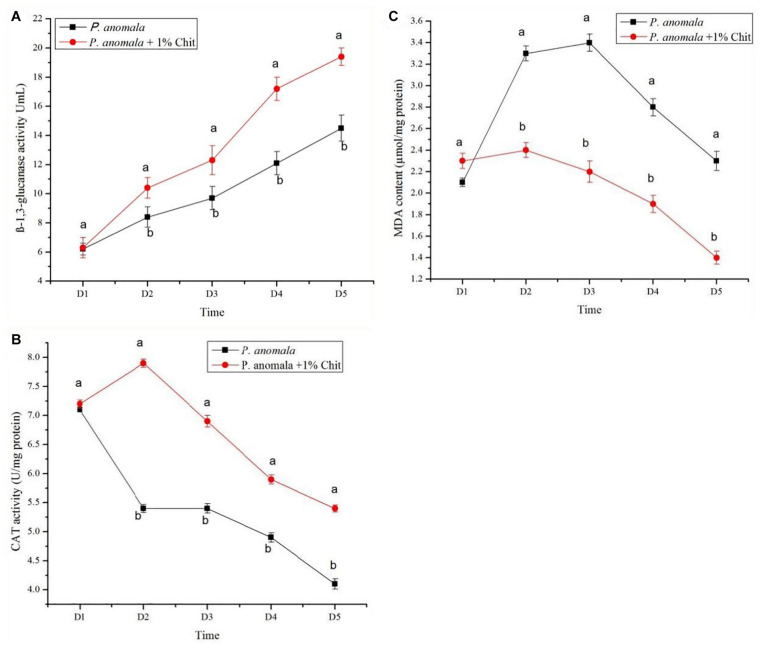
The β-1,3-glucanase activity, CAT, and MDA content of *P. anomala* induced with 1% chitosan for 5 days are shown in Figure. **(A)** The activity of β-1,3-glucanase increased with the storage time. A higher result was recorded for yeast incubated with chitosan as comparedto the control. Incubation of *P. anomala* with 1% chitosan also significantly induced the activity of CAT as comparedto the control **(B)**. Even though the initial MDA accumulationof the yeast was the same, it showed significantly lower results for the yeast incubated with 1% chitosan **(C)**.

The β-1,3-glucanase activity, CAT, and MDA content of *P. anomala* induced with 1% chitosan for 5 days are shown in Figure 1. As shown in the figure, the activity of β-1,3-glucanase increased with the storage time. A higher result was recorded for yeast incubated with chitosan as compared to the control. Incubation of *P. anomala* with 1% chitosan also significantly induced the activity of CAT as compared to the control. Even though the initial MDA accumulation of the yeast was the same, it showed significantly lower results for the yeast incubated with 1% chitosan. These results confirm that chitosan (1% w/v) can enhance the bio-control efficacy of *P. anomala*.

### Analysis of Table Grapes Defense-Related Enzyme Activities After Being Treated by *P. anomala* Induced With Chitosan (1% w/v)

#### Polyphenoloxidase (PPO) Activity

**FIGURE 2:**
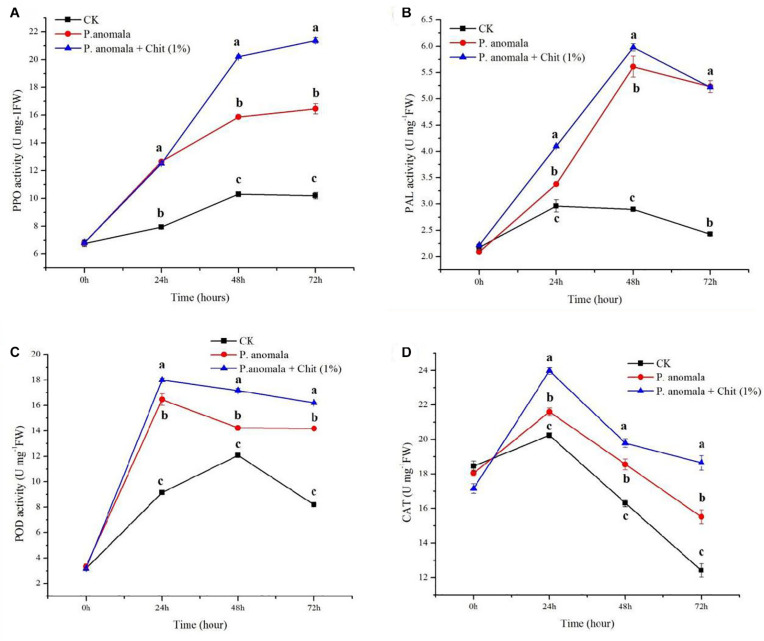
Time course change of disease defense-related enzyme activities in grapes incubated at 20°C and 95% R.H for 3 days **(A)** PPO, **(B)** PAL, **(C)** POD, and **(D)** CAT. Treatments were as follows: **(A)** Control (double distilled water), **(B)** *P. anomala* alone (1 × 10^8^), and **(C)** *P. anomala* enhanced with chitosan (1% w/v). Each treatment was replicated three times, and values with different letters are significantly different according to Tukey’s mean comparison (*p* < 0.05) test.

The PPO activity of grapes treated with *P. anomala* and *P. anomala* supplemented with chitosan (1% w/v) stored at 20°C is shown in Figure 2A. In the first 24 h, there was no significant difference between *P. anomala* alone, and *P. anomala* supplemented with chitosan (1% w/v). However, after 24 h *P. anomala* supplemented with chitosan (1% w/v) showed the highest PPO activity compared to the other two treatments. The control group showed the lowest activity throughout the storage period. This showed that the yeast could initiate PPO activity in grapes.

#### Phenylalanine Ammonia Lyase (PAL) Activity

*P. anomala* alone and *P. anomala* supplemented with chitosan (1% w/v) induced the PAL activity during the first 2 days of the storage period and decline after decline on day 3 (Figure 2B). PAL activity in grapes was highly induced for those treated by *P. anomala* supplemented with chitosan (1% w/v) compared to grapes treated with *P. anomala* alone the first 2 days. However, the PAL activity showed a similar result after 3 days of storage at 20°C. The control group showed the lowest PAL activity throughout the storage period. These suggest that *P. anomala* has a significant effect on inducing PAL activity during post-harvest storage.

#### Peroxidase (POD) Activity

The POD activity of grapes significantly increased in both treatments than the control during the storage period at 20°C (Figure 2C). For grapes treated with *P. anomala* alone and *P. anomala* supplemented with chitosan (1% w/v), the enzyme activity sharply increased on the first storage day and then declined. However, the control group showed an increase in POD activity until 48 h and then sharply declined. The control group showed the lowest enzyme activity, and grapes treated with *P. anomala* were supplemented with chitosan (1% w/v) and showed the highest enzyme activity throughout the storage period.

#### Catalase (CAT) Activity

The CAT activity of grapes treated with *P. anomala* alone and *P. anomala* supplemented with chitosan (1% w/v) is shown in Figure 2D. The CAT activity increased during the first day of storage and started to decline after 24 h of storage. The control group showed the lowest enzyme activity, and grapes treated with *P. anomala* supplemented with chitosan (1% w/v) showed the highest enzyme activity throughout the storage period. After 72 h of storage, the CAT activity of grapes treated with *P. anomala* alone and the control group showed below the initial CAT activity (17 U mg/FW).

### Effect *P. anomala* Induced With Chitosan on Defense-Related Enzyme Genes Expression Levels of Table Grapes

**FIGURE 3:**
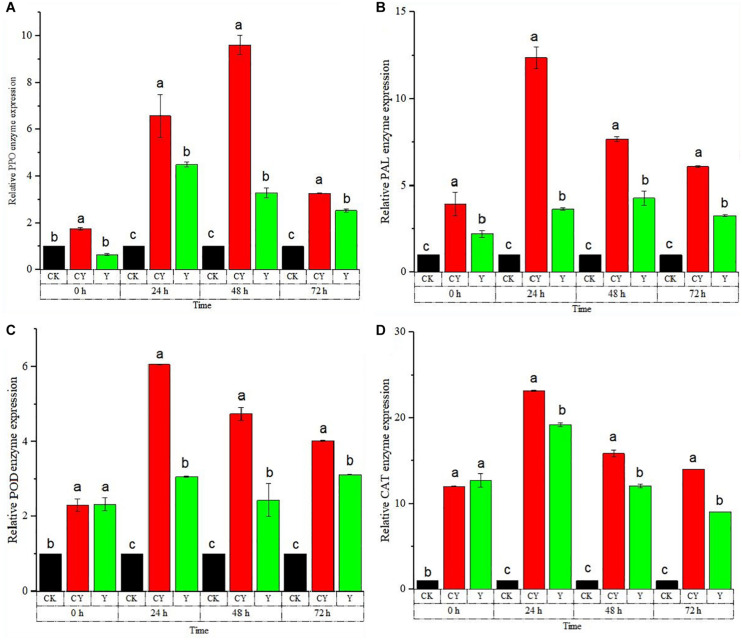
Time course change of defense-related genes expression in table grapes. **(A)** polyphenol oxidase (*PPO*), **(B)** phenylalanine ammonia-lyase (*PAL*), **(C)** peroxidase (POD), and **(D)** catalase (*CAT*) enzymes. The results were obtained using RT-qPCR to determine relative gene expression levels of table grape berries incubated at 20°C from 0 to 72 h. Treatments: CK (Control) (sterile distilled water), CY (yeast incubated in 1% chitosan), and Y (Yeast alone). The results represent the mean of two independent experiments, each treatment with three replicates. Different letters on each bar indicate statistical significance at *p* < 0.05 using Tukey’s mean separation test. 0 h means 1 h after the treatments were carried out.

The RT-qPCR experiment was conducted to confirm the reliability of the activity of the defense-related enzymes discussed in section “Analysis of Table Grapes Defense-Related Enzyme Activities After Being Treated by *P. anomala* Induced With Chitosan (1% w/v).” The RT-qPCR of the five major defense-related enzymes shown in Figure 3. As shown in Figure 3A, the PPO enzyme gene expression was highest for table grapes treated with chitosan induced with chitosan (1% w/v) throughout the storage period.

On the second storage day, the highest expression level of PPO was recorded as around 9.45 fold that of the control group. The relative gene expression of the APX enzyme sharply rises the next day soon after storage, where it peaked 48 h after storage. The gene expression then started falling down the next day.

Throughout the storage period, grapes treated with *P. anomala* induced with chitosan showed the highest APX gene expression, which is a confirmatory finding for the previous results. The CHI enzyme gene expression is shown in Figure 3C. As shown in the figure, tables grapes treated with *P. anomala* induced with chitosan (1% w/v) showed the highest expression level compared to the yeast alone and the control group. The highest CHI expression was recorded 24 h after storage, which was about 4.05 fold of the control group.

On the third and fourth days, the CHI gene expression of table grapes treated with *P. anomala* was the same as the control group even though it showed a higher result on the second day. PAL enzyme’s relative gene expression sharply rises the next day soon after storage, where it reached its peak point on the third day after storage. Throughout the storage period, grapes treated with *P. anomala* induced with chitosan (1% w/v) showed the highest PAL gene expression, which is a confirmatory finding for the previous results. The CAT enzyme’s relative gene expression sharply rose, where it showed the highest results for table grapes treated with *P. anomala* induced with chitosan (1% w/v). The highest result was seen on the third day after storage for table grapes treated with *P. anomala* induced with chitosan, which was about 23.5 fold that of the control group. Throughout the storage period, grapes treated with *P. anomala* induced with chitosan showed the highest CAT gene expression, which is a confirmatory finding for the previous results.

In general, the above defense-related enzyme gene expression levels of table grapes were significantly up-regulated by *P. anomala* induced with chitosan (1% w/v), and the results verified the accuracy of enzyme activity experiments in section “Analysis of Table Grapes Defense-Related Enzyme Activities After Being Treated by *P. anomala* Induced With Chitosan (1% w/v).”

### Non-enzymatic Assays

#### Total Phenolic Content

**FIGURE 4:**
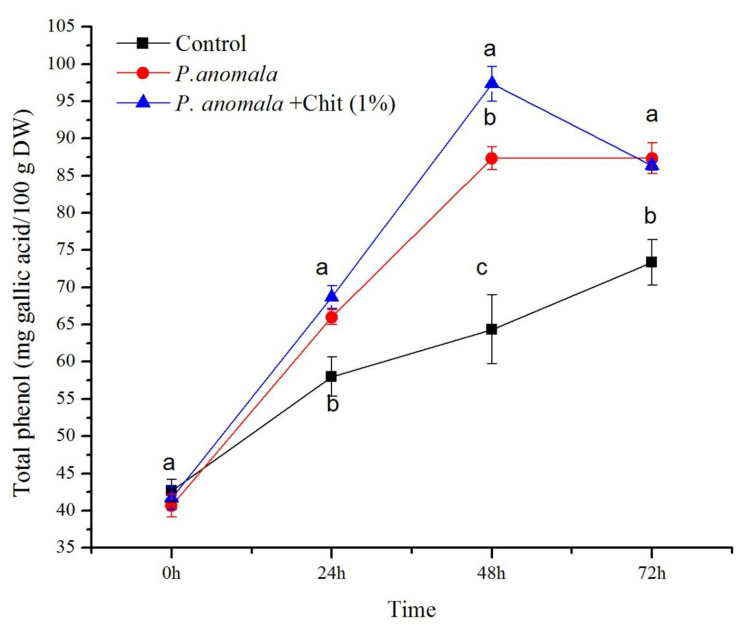
Time course change of phenol content (mg gallic acid equivalents/100 g DW) extracts from table grape stored at 20°C. Treatments: CK (Control) (sterile distilled water), CY (*P. anomala* incubated in 1% chitosan), and Y (*P. anomala* alone). The results represent the mean of two independent experiments, each treatment with three replicates. Different letters on each bar indicate statistical significance at *p* < 0.05 using Tukey’s mean separation test. 0 h means 1 h after the treatments were carried out.

The total phenolic content of table grapes treated with *P. anomala* or *P. anomala* induced with chitosan (1% w/v) is shown in Figure 4. Grapes treated with *P. anomala* induced with chitosan showed the highest total phenolic content (ranged between 41.67 and 97.33 mg of gallic acid equivalents/100 g DW (GAE/100 g DW)) during the storage period. The highest total phenolic content was recorded at 48 h post-treatment, which was more than 1.5 fold than the control group. Grape tissue without any treatment (the control) showed the lowest total phenolic content (ranging between 42.67 and 73.33 GAE/100 g DW) during the storage period.

#### Total Flavonoid Content (TFC)

**FIGURE 5:**
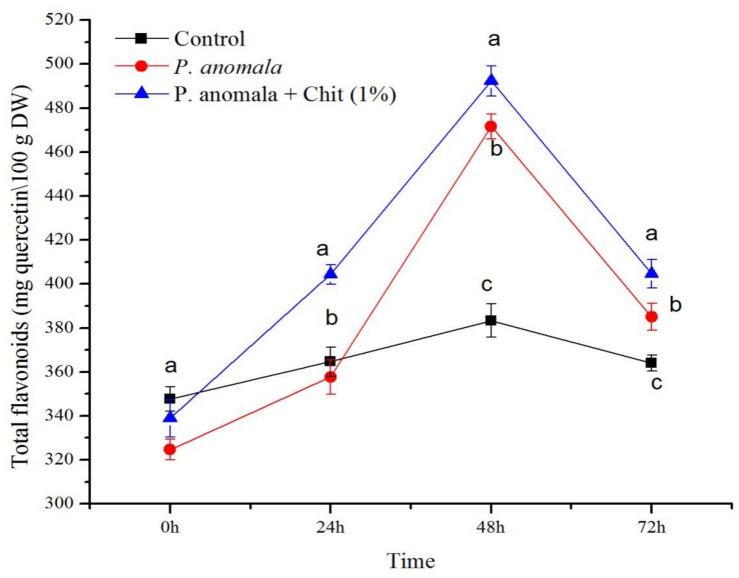
Time course of flavonoid content (mg quercetin equivalents/100 g DW) extracts from table grape stored at 20°C. Treatments: CK (Control) (sterile distilled water), CY (*P. anomala* incubated in 1% chitosan), and Y (*P. anomala* alone). The results represent the mean of two independent experiments, each treatment with three replicates. Different letters on each bar indicate statistical significance at *p* < 0.05 using Tukey’s mean separation test. 0 h means 1 h after the treatments were carried out.

The TFC of grape tissues at a different time interval during the storage period is shown in Figure 5. Comparing the grape tissues treated with *P. anomala* induced with chitosan (1% w/v) with the control group, the TFC of the tissues treated with the yeast showed the highest (and ranged between 339 and 404.33 mg of quercetin equivalents/100 g DW (QE/100 g DW)) during the storage period. The highest TFC was recorded 48 h post-treatment, which was more than 1.02 fold compared to the control group. Grape tissues without any treatment (the control group) showed the lowest TFC (ranging between 347.67 and 364.67 QE/100 g DW) during the storage period.

## Discussion

Our previous research investigated whether chitosan (1% w/v) enhanced the bio-control efficacy of *P. anomala* against *P. expansum* both *in vitro* and *in vivo* (Godana et al., 2020). In the current study, we were interested in the mechanisms by which chitosan improved the bio-control efficacy of *P. anomala* against *P. expansum* in table grapes. The mechanism by which *P. anomala* suppresses the growth of *P. expansum* in the fruit is crucial for replacing the antagonistic yeasts with commercial fungicides.

The current study showed that incubation of *P. anomala* with 1% chitosan significantly increased the enzymatic activities of yeasts such as β-1,3-glucanase and CAT; and reduced the accumulation of MDA. Enzymes such as β-1,3-glucanase were found to increase the antagonistic efficacy of yeasts. In addition, the inducible enzyme degrades the cell wall of pathogens and leads to cell lysis (Dahiya et al., 2006; Kang, 2012). Incubation of *P. anomala* with chitosan (1% w/v) also induced the CAT enzymatic activity, an enzyme that was determined to enhance the bio-control efficacy of other yeasts as well (Zhao et al., 2018). The CAT was found to be a good detoxification agent when plants are injured and accumulate a large number of reactive oxygen species (ROS) at the wound site, which leads to cell damage (An et al., 2012). The lowering of MDA content also lowers the oxidative damage to the yeast cells. The current result confirmed that the MDA content of the yeast cells incubated with 1% chitosan is lower compared to the control which improves the yeast bio-control performance.

When plants are attacked by pathogens, they enhance their defense mechanism through a broad spectrum of methods. Among them, the production of phenolic compounds, phytoalexins, and pathogenesis-related (PR) proteins helps to prevent pathogen invasion (Bowles, 1990). Pathogenesis-related proteins are disease defense enzymes that help control the rate of disease spread during infection (Deborah et al., 2001; Kumari and Vengadaramana, 2017). PAL, PPO, POD, and CAT are among the four crucial disease defense enzymes, and their activities are positively correlated with fruit disease resistance (Qin et al., 2015; Zhang et al., 2016; Youssef et al., 2020).

PPO can produce antimicrobial phenolic substances by the metabolism of phenols and oxidizing phenolic compounds into toxic quinones (Qin et al., 2015). Thus, it has a vital role in host plant defense mechanisms. Mahunu et al. (2016) reported that the increase in PPO activity reduced apples’ blue mold disease. The current research also confirms that the use of *P. anomala* induced with chitosan significantly increased the PPO activity of table grapes, which significantly contribute to reduce the blue mold disease of table grapes caused by *P. expansum*.

PAL is a key and rate-limiting enzyme during the shikimic acid pathway in the phenolic metabolism during plant disease resistance. It plays a crucial role during lignin synthesis and accumulation. PAL is also actively involved in salicylic acid’s biosynthesis, a hormone required for plant defense (Qin et al., 2015). PAL is responsible for the biosynthesis of p-coumaric acid derivatives, phytoalexin, and lignins that contribute to plant defense systems (Qin et al., 2003). In addition, it participates in the biosynthesis of the defense hormone salicylic acid, which is required for both local and systemic acquired resistance in plants (Dixon and Paiva, 1995).

Thus, the activity of PAL has a positive correlation with plant disease resistance. Our study investigated that the treatment of table grapes with *P. anomala* induced with chitosan (1% w/v) increased PAL activity. This shows that the antagonistic yeast suppresses the fungal growth by up-regulating the fruit disease defense enzymes and direct parasitism. Li and Tian (2006) also reported that the antagonistic yeast *Cryptococcus laurentii* up-regulated the PAL activity in the apple, which controlled the blue mold disease of the fruit.

Stimulation of ROS makes plant tissues vulnerable to fungal attack. POD and CAT are the main protective enzymes against ROS in plant tissues. POD is also noted to control the balance of H_2_O_2_ in the fruit’s cell wall, which is essential for the cross-linking of phenolic compounds in response to several external stress factors such as injuries, pathogen attack, and environmental stress (Passardi et al., 2004). In our study, the treatment of grapes with *P. anomala* induced with chitosan (1% w/v) significantly increased both POD and CAT activity.

The RT-qPCR enables the reliable detection and measurement of the five main disease defense enzymes (PPO, PAL, POD, CHI, and CAT). Our study confirmed that treating table grapes with the yeast *P. anomala* alone or enhanced with chitosan up-regulated these enzymes. These further confirm that *P. anomala* alone or induced with chitosan (1% w/v) activate the defense mechanism of table grapes, up-regulate the expression of defense genes, and synthesize many other corresponding defense enzymes, thus improving the defense ability of table grapes against diseases.

Phenolic compounds are among the most important secondary metabolites that act as antioxidants in table grapes. They can strongly affect berry quality (color, flavor, astringency, and bitterness) and functional properties (Chamkha et al., 2003). Phenolic compounds can also increase the antimicrobial properties of table grapes (Champa et al., 2015). Raimbault et al. (2010) reported that higher enzymatic activity in pineapples leads to higher phenolic compound concentration during post-harvest storage. The availability of hydrogen peroxide, which is majorly affected by the POD enzyme, also affects the phenolic groups’ cross-linking response to different external stresses (Passardi et al., 2004). In the current study, table grapes treated with *P. anomala* induced with chitosan showed the highest POD activity and highest total phenolic compound concentration, which supports previous studies by other researchers, which contribute to the fruit post-harvest shelf and help to mitigate both biotic and abiotic stresses during post-harvest storage. Al-Qurashi and Awad (2015) stated that table grapes’ post-harvest treatment with 1% chitosan could increase the total phenolics and flavonoid level and increase POD activity. Higher accumulation of phenolic compounds in the fruit may also occur because of an increment of PAL enzyme when plants are exposed to biotic and abiotic stress such as high temperature, fungi, bacterial, and virus infections (Solecka and Kacperska, 2003).

Flavonoids are polyphenolic secondary metabolites that play different roles in many physiological activities, acting as phytoalexins and phytoanticipins in plant defense pathogens (Desta et al., 2016). In addition to their antimicrobial characteristics, flavonoids help our health during consumption and can reduce the risk of cancer, neurodegenerative diseases, heart disease, inflammation, aging, arthritis, and diabetes (Kumar and Pandey, 2013). The current study also confirms that post-harvest treatment of table grapes with *P. anomala* induced with chitosan can significantly increase the accumulation of total flavonoids in the fruit, which has a significant role, enabling the fruit to combat post-harvest diseases and other biotic and abiotic stresses.

## Conclusion

Post-harvest treatment of table grapes with the antagonistic yeast *P. anom*aly alone or induced with chitosan (1% w/v) enhance the content and activities of PPO, POD, PAL, CAT, total phenol, and flavonoid compounds. These findings contribute to knowledge of the mechanisms by which the antagonistic yeast enhances resistance against blue mold disease in table grapes. From the current study, it can be concluded that the yeast *P. anomala* enhanced with chitosan induced host resistance, which is directly related to enhancing the antioxidant system. This study will further enable future detailed investigation in the yeast pathogen control mechanisms to use yeasts as bio-pesticides.

## Data Availability Statement

The original contributions presented in the study are included in the article/supplementary material, further inquiries can be directed to the corresponding author.

## Author Contributions

EG: writing – original draft, methodology, data curation, and formal analysis. QY, XZ, and LZ: resources, supervision, validation, and visualization. HZ: funding acquisition, project administration, supervision, validation, writing – review, and editing. JL: methodology, writing – review, and editing. All authors contributed to the article and approved the submitted version.

## Conflict of Interest

The authors declare that the research was conducted in the absence of any commercial or financial relationships that could be construed as a potential conflict of interest.

## Abbreviations

CAT: catalase
MDA: malondialdehyde
PPO: Polyphenol oxidase
PAL: phenylalanine
POD: peroxidase.

## References

[B1] Abdelhai M. H. Awad F. N. Yang Q. Mahunu G. K. Godana E. A. Zhang H. (2019). Enhancement the biocontrol efficacy of *Sporidiobolus pararoseus* Y16 against apple blue mold decay by glycine betaine and its mechanism. *Biol. Control.* 139:104079. 10.1016/j.biocontrol.2019.104079

[B2] Abo-Elyousr K. A. M. Al-Qurashi A. D. Almasoudi N. M. (2021). Evaluation of the synergy between *Schwanniomyces vanrijiae* and propolis in the control of *Penicillium digitatum* on lemons. *Egypt. J. Biol. Pest Control* 31:66. 10.1186/s41938-021-00415-4

[B3] Al-Qurashi A. D. Awad M. A. (2015). Postharvest chitosan treatment affects quality, antioxidant capacity, antioxidant compounds and enzymes activities of ‘El-Bayadi’ table grapes after storage. *Sci. Hortic*. 197 392–398. 10.1016/j.scienta.2015.09.060

[B4] An B. Li B. Qin G. Tian S. (2012). Exogenous calcium improves viability of biocontrol yeasts under heat stress by reducing ROS accumulation and oxidative damage of cellular protein. *Curr. Microbiol*. 65 122–127. 10.1007/s00284-012-0133-4 22562600

[B5] Aquino-Bolaños E. N. Mercado-Silva E. (2004). Effects of polyphenol oxidase and per oxidase activity, phenolics and lignin content on the browning of cut jicama. *Postharvest Biol. Technol*. 33 275–283. 10.1016/j.postharvbio.2004.03.009

[B6] Bowles D. J. (1990). Defense-related proteins in higher plants. *Annu. Rev. Biochem*. 59 873–907. 10.1146/annurev.bi.59.070190.004301 2197993

[B7] Boysen M. E. Stina B. Johan S. (2000). Effect of the biocontrol yeast *Pichia anomala* on interactions between *Penicillium roqueforti*, *Penicillium carneum*, and *Penicillium paneum* in moist grain under restricted air supply. *Postharvest Biol. Technol*. 19 173–179. 10.1016/S0925-5214(00)00086-7

[B8] Chamkha M. Cathala B. Cheynier V. Douillard R. (2003). Phenolic Composition of Champagnes from Chardonnay and Pinot Noir Vintages. *J. Agric. Food Chem*. 51 3179–3184. 10.1021/jf021105j 12720412

[B9] Champa W. A. H. Gill M. I. S. Mahajan B. V. C. Arora N. K. (2015). Preharvest salicylic acid treatments to improve quality and postharvest life of table grapes (*Vitis vinifera L*.) cv. Flame Seedless. *J. Food Sci. Technol*. 52 3607–3616. 10.1007/s13197-014-1422-7 26028743 PMC4444924

[B10] Dahiya N. Tewari R. Hoondal G. S. (2006). Biotechnological aspects of chitinolytic enzymes: a review. *Appl. Microbiol. Biotechnol*. 71 773–782. 10.1007/s00253-005-0183-7 16249876

[B11] de Oliveira V. C. E. Magnani M. de Sales C. V. Lima de Souza Pontes A. Campos -Takaki G. M. Montenegro Stamford T. C. (2014). Effects of post -harvest treatment using chitosan from *Mucor circinelloides* on fungal pathogenicity and quality of table grapes during storage. *Food Microbiol.* 44 211–219. 10.1016/j.fm.2014.06.007 25084665

[B12] Deborah S. D. Palaniswami A. Vidhyasekaran P. Velazhahan R. (2001). Time-course study of the induction of defense enzymes, phenolics and lignin in rice in response to infection by pathogen and non-pathogen. *J. Plant Dis. Prot*. 108 204–216.

[B13] Desta K. T. Shin S. C. Shim J. H. Kim G. S. Shin H. C. El-Aty A. M. A. (2016). Flavonoid variations in pathogen-infected plants. *Front. Nat. Prod. Chem.* 2 393–439. 10.2174/9781681083599116020009

[B14] Di Francesco A. Martini C. Mari M. (2016). Biological control of postharvest diseases by microbial antagonists: how many mechanisms of action? *Eur. J. Plant Pathol*. 145 711–717. 10.1007/s10658-016-0867-0

[B15] Dixon R. A. Paiva N. L. (1995). Stress-induced phenylpropanoid metabolism. *Plant Cell* 7 1085–1097. 10.2307/3870059 12242399 PMC160915

[B16] Elisabeth F. Ulrika D. Marianne E. B. Karl-Johan L. Johan S. (2002). Physiological characteristics of the biocontrol yeast *Pichia anomala* J121. *FEMS Yeast Res*. 2 395–402. 10.1016/S1567-1356(02)00098-3 12702290

[B17] Franck J. Latorre B. A. Torres R. Zoffoli J. P. (2005). The effect of preharvest fungicide and postharvest sulfur dioxide use on postharvest decay of table grapes caused by *Penicillium expansum*. *Postharvest Biol. Technol*. 37 20–30. 10.1016/j.postharvbio.2005.02.011

[B18] Godana E. A. Yang Q. Wang K. Zhang H. Zhang X. Zhao L. (2020). Bio-control activity of *Pichia anomala* supplemented with chitosan against *Penicillium expansum* in postharvest grapes and its possible inhibition mechanism. *LWT Food Sci. Technol*. 124:109188. 10.1016/j.lwt.2020.109188

[B19] Gurnani N. Gupta M. Mehta D. Mehta B. K. (2016). Chemical composition, total phenolic and flavonoid contents, and in vitro antimicrobial and antioxidant activities of crude extracts from red chilli seeds (*Capsicum frutescens L*.). *J*. *Taibah Univ*. *Sci*. 10 462–470. 10.1016/j.jtusci.2015.06.011

[B20] Madbouly A. Abo Elyousr K. A. M. Mohamed Ismail I. (2020). Biocontrol of *Monilinia fructigena*, causal agent of brown rot of apple fruit, by using endophytic yeasts. *Biol. Control* 144:104239. 10.1016/j.biocontrol.2020.104239

[B21] Kang T. (2012). Biological control of postharvest diseases of fruits and vegetables by microbial antagonists. *J. Nanjing Agric. Univ.* 40 411–441. 10.1146/annurev.phyto.40.120401.130158 12147766

[B22] Kumar S. Pandey A. K. (2013). Chemistry and Biological Activities of Flavonoids: an Overview. *Sci. World J*. 2013:162750. 10.1155/2013/162750 24470791 PMC3891543

[B23] Kumari Y. S. Vengadaramana A. (2017). Stimulation of Defense Enzymes in Tomato (*Solanum lycopersicum L.)* and Chilli (*Capsicum annuum L.*) in Response to Exogenous Application of Different Chemical Elicitors. *Univers. J. Plant Sci*. 5 10–15. 10.13189/ujps.2017.050102

[B24] Li B. Tian S. (2006). Effects of trehalose on stress tolerance and biocontrol efficacy of *Cryptococcus laurentii*. *J. Appl. Microbiol*. 100 854–861. 10.1111/j.1365-2672.2006.02852.x 16553742

[B25] Livak K. J. Schmittgen T. D. (2001). Analysis of Relative Gene Expression Data Using Real- Time Quantitative PCR and the 2^–ΔΔCT^ Method. *Methods* 25 402–408. 10.1006/meth.2001.1262 11846609

[B26] Lurie S. Fallik E. Handros A. Shapira R. (1997). The possible involvement of peroxidase in resistance to *Botrytis cinerea* in heat treated tomato fruit. *Physiol*. *Mol*. *Plant Pathol*. 50 141–149. 10.1006/pmpp.1996.0074

[B27] Mahunu G. K. Zhang H. Yang Q. Zhang X. Li D. Zhou L. (2016). Improving the biocontrol efficacy of *Pichia caribbica* with phytic acid against postharvest blue mold and natural decay in apples. *Biol. Control.* 92 172–180. 10.1016/j.biocontrol.2015.10.012

[B28] Passardi F. Penel C. Dunand C. (2004). Performing the paradoxical: how plant peroxidases modify the cell wall. *Trends Plant Sci*. 9 534–540. 10.1016/j.tplants.2004.09.002 15501178

[B29] Plata C. Millan C. Mauricio J. C. Ortega J. M. (2003). Formation of ethyl acetate and isoamyl acetate by various species of wine yeasts. *Food Microbiol.* 20 217–224. 10.1016/S0740-0020(02)00101-6

[B30] Qin G. Z. Tian S. P. Xu Y. Wan Y. L. (2003). Enhancement of biocontrol efficacy of antagonistic yeasts by salicylic acid in sweet cherry fruit. *Physiol. Mol. Plant Pathol*. 62 147–154. 10.1016/S0885-5765(03)00046-8

[B31] Qin X. Xiao H. Xue C. Yu Z. Yang R. Cai Z. (2015). Biocontrol of Gray Mold in Grapes with the Yeast *Hanseniaspora uvarum* alone and in Combination with Salicylic Acid or Sodium Bicarbonate. *Postharvest Biol. Technol*. 100 160–167. 10.1016/j.postharvbio.2014.09.010

[B32] Raimbault A. K. Marie-Alphonsine P. A. Horry J. P. Francois-Haugrin M. Romuald K. Soler A. (2010). Polyphenol oxidase and peroxidase expression in four pineapple varieties (*Ananas comosus L*.) after a chilling injury. *J. Agric. Food Chem*. 59 342–348. 10.1021/jf102511z 21133422

[B33] Renouf V. Claisse O. Lonvaud-Funel A. (2007). Inventory and monitoring of wine microbial consortia. *Appl. Microbiol. Biotechnol*. 75 149–164. 10.1007/s00253-006-0798-3 17235561

[B34] Sanzani S. M. Montemurro C. Di Rienzo V. Solfrizzo M. Ippolito A. (2013). Genetic structure and natural variation associated with host of origin in *Penicillium expansum* strains causing blue mould. *Int. J. Food Microbiol*. 165 111–120. 10.1016/j.ijfoodmicro.2013.04.024 23728428

[B35] Sanzani S. M. Reverberi M. Punelli M. Ippolito A. Fanelli C. (2012). Study on the role of patulin on pathogenicity and virulence of *Penicillium expansum*. *Int. J. Food Microbiol*. 153 323–331. 10.1016/j.ijfoodmicro.2011.11.021 22189024

[B36] Sawicka B. (2019). “Post-harvest Losses of Agricultural Produce,” in *Zero Hunger, Encyclopedia of the UN Sustainable Development Goals*, eds Leal Filho W. Azul A. Brandli L. Özuyar P. Wall T. (Cham: Springer). 10.1007/978-3-319-69626-3_40-1

[B37] Solecka D. Kacperska A. (2003). Phenylpropanoid deficiency affects the course of plant acclimation to cold. *Physiol. Plant*. 119 253–262. 10.1034/j.1399-3054.2003.00181.x 11841302

[B38] Spadaro D. Droby S. (2015). Unraveling the mechanisms used by antagonistic yeast to control postharvest pathogens on fruit. *Acta Hortic.* 1144 63–70. 10.17660/actahortic.2016.1144.9

[B39] Swain T. Hillis W. E. (1959). The phenolic constituents of *Prunus domestica*. I. - The quantitative analysis of phenolic constituents. *J. Sci. Food Agric*. 10 63–68. 10.1002/jsfa.2740100110

[B40] Vázquez G. Fontenla E. Santos J. Freire M. S. González-Álvarez J. Antorrena G. (2008). Antioxidant activity and phenolic content of chestnut (*Castanea sativa*) shell and eucalyptus (*Eucalyptus globulus*) bark extracts. *Ind Crops Prod.* 28 279–285. 10.1016/j.indcrop.2008.03.003

[B41] Wang M. Zhao L. Zhang X. Dhanasekaran S. Abdelhai M. H. Yang Q. (2019). Study on biocontrol of postharvest decay of table grapes caused by *Penicillium rubens* and the possible resistance mechanisms by *Yarrowia lipolytica*. *Biol. Control* 130 110–117. 10.1016/j.biocontrol.2018.11.004

[B42] Wang Y. Li Y. Xu W. Zheng X. Zhang X. Abdelhai M. H. (2018). Exploring the effect of β-glucan on the biocontrol activity of *Cryptococcus podzolicus* against postharvest decay of apples and the possible mechanisms involved. *Biol. Control* 121 14–22. 10.1016/j.biocontrol.2018.02.001

[B43] Wang Y. S. Tian S. P. Xu Y. Qin G. Z. Yao H. (2004). Changes in the activities of proand anti-oxidant enzymes in peach fruit inoculated with *Cryptococcus laurentii* or *Penicillium expansum* at 0 or 20°C. *Postharvest Biol. Technol*. 34 21–28. 10.1016/j.postharvbio.2004.04.003

[B44] Wisniewski M. Droby S. Norelli J. Liu J. Schena L. (2016). Alternative management technologies for postharvest disease control: the journey from simplicity to complexity. *Postharvest Biol. Technol*. 122 3–10. 10.1016/j.postharvbio.2016.05.012

[B45] Youssef K. Roberto S. R. Tiepo A. N. Constantino L. V. de Resende J. T. V. Abo-Elyousr K. A. M. (2020). Salt Solution Treatments Trigger Antioxidant Defense Response against Gray Mold Disease in Table Grapes. *J. Fungi* 6:179. 10.3390/jof6030179 32962077 PMC7558686

[B46] Zhang C. Chen K. Wang G. (2013). Combination of the biocontrol yeast *Cryptococcus laurenti*i with UV-C treatment for control of postharvest diseases of tomato fruit. *Biocontrol* 58 269–281. 10.3390/toxins9020066 28216565 PMC5331445

[B47] Zhang X. Li Y. Wang H. Gu X. Zheng X. Wang Y. (2016). Screening and Identification of Novel Ochratoxin A-Producing Fungi from Grapes. *Toxins* 8:333. 10.3390/toxins8110333 27845758 PMC5127129

[B48] Zhao L. Sun Y. Yang D. Li J. Gu X. Zhang X. (2018). Effects of *Sporidiobolus pararoseus* Y16 on postharvest blue mold decay and the defense response of apples. *J. Food Qual*. 2018 1–9. 10.1155/2018/6731762

[B49] Zhao Z. Zhang H. Li J. Cui J. Zhang X. Ren X. (2012). Enhancement of biocontrol efficacy of *Pichia carribbica* to postharvest diseases of strawberries by addition of trehalose to the growth medium. *Int*. *J*. *Mol*. *Sci*. 13 3916–3932. 10.3390/ijms13033916 22489189 PMC3317749

[B50] Zoffoli J. P. Latorre B. A. (2011). “Table grape (*Vitis vinifera* L.),” in *Postharvest Biology and Technology of Tropical and Subtropical Fruits: Cocona to Mango*, ed. Yahia E. M. (Oxford: Woodhead Publishing), 179–214. 10.1533/9780857092885.179

